# Inequities in eye care in South Asia

**Published:** 2017-02-10

**Authors:** Thulasiraj Ravilla

**Affiliations:** 1 Executive Director- Aravind Eye Care Systems

Inequities are often discovered and discussed around prevalence studies, which produces data relating to various types of inequities. It sits in the space of periodic assessment and continues to remain predominantly in the knowledge rather than action realm. Therefore there is a need fora paradigm shift in how we think about and approach inequities in service. The goal of “VISION 2020 - The Right to Sight” and that of many organizations and governments engaged in eye care is around “eliminating avoidable blindness”. This implicitly means that there are people who are blind, but don't need to be. This is true since proven interventions exist to treat or prevent the major causes of blindness or visual impairment.

Globally, it is reported that 39 million people are blind and a further 246 million have moderate or severe visual impairment.^1^ A significant number of people with avoidable visual loss are not being reached and served by the current eye care delivery system for a variety of reasons including patient awareness and access to services. Thus, we need to recognize that inequity in the eye care delivery system is a significant cause of the remaining problem of avoidable blindness. Therefore it is relevant to look at how we currently provide eye care and redesign it with an explicit focus on the goal of eliminating inequities. Such a health system design should have inbuilt, on-going monitoring and processes for continuously identifying and correcting inequities as they occur, similar to what is done in clinical audit and care process for reducing complications, infections, etc. It is time that this paradigm shift occurs in the design of eye care services both at institutional and national level. In order to consider the redesign of eye care delivery, it is important to have an understanding of the origin of these inequities. Overall, inequity or those not served fall into a few broad categories:

## Inequity due to socio-economic factors:

These relate to gender, literacy, marital status and wealth. They influence individuals on the level of empowerment, awareness, decision making position in the family, priority for eye care and the extent of their mobility. Several studies undertaken in this region have shown a strong association between cataract blindness and these factors. Studies in India have shown that women have a 20% higher chance of being blind than men; illiterate people are 3.7 times more likely to be blind than people who are literate; and unemployed people are twice as likely to be blind than employed people.^2^ Similarly studies in Bangladesh have shown that married persons are almost half OR = 0.6 (0.4 − 0.9) as likely to be blind as single / widowed persons.^3^

**Figure:**
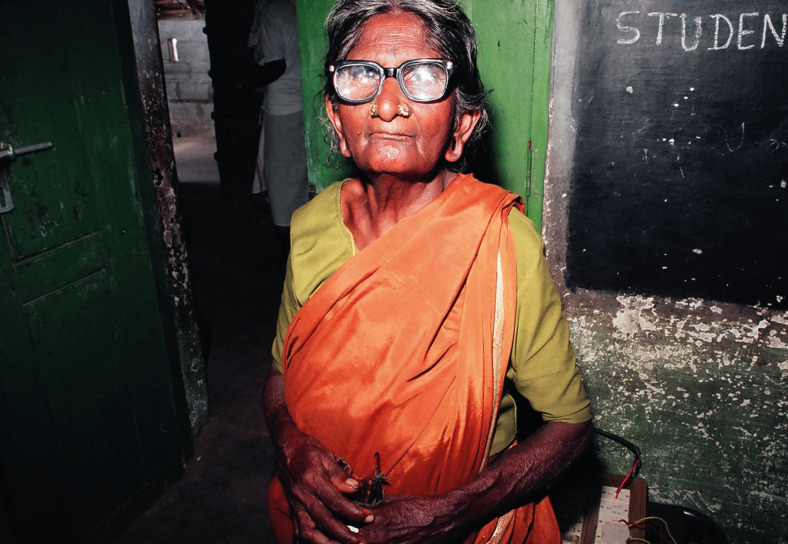
Inequity in eye care is one of the primary reasons for the continuing problem of avoidable blindness.

**Table – 1:** Socio-demographic correlates of cataract blindnes

Socio-demographic variables	Adjusted Odds Ratio	95% CI
**Female**	1.2	(1.2-1.3)
**Rural**	1.2	(1.1-1.4)
**Illiterate**	3.7	(2.7-5.2)
**Not Working**	2.0	(1.8-2.2)

**Source: Current estimates of blindness in India; BJO 2005;89;257–260**

## Location:

Which same study? showed that those living in rural areas have a slightly increased risk 1.2 (1.1 – 1.4) of blindness over those living in urban situations.

The locational disadvantage that we see at the individual level also plays out at national level. In the more affluent or developed countries, the overall prevalence of blindness is lower and those blind due to avoidable causes are much less. This will reduce even further with the advent of emerging treatment for conditions like DR, ARMD and Glaucoma. In contrast if we look at low income countries preventable causes of blindness due to trachoma, vitamin-A deficiency and onchocerciasis still occur in some poor communities and blindness due to treatable cataract is still the major cause.

## Disease focus:

In design of services and interventions, unconsciously or sometimes due to the purpose of funding (as in the World Bankfunded cataract programme in India), the focus tends to be on certain conditions. At individual or institutional provider level such focus emerges often on account of economic considerations. For instance, in most of the countries in this region and developing countries in general, the overarching focus has been on cataract blindness and provision of cataract surgery.

While this has made an impact on cataract blindness it has also led to a clinical practice which is not comprehensive and people with other conditions, as simple as refractive errors or with complex retinal pathologies have not received equitable attention. Thus the biased preference to some conditions has contributed in its own way to inequity in the treatment and the management of other treatable conditions.

**Figure:**
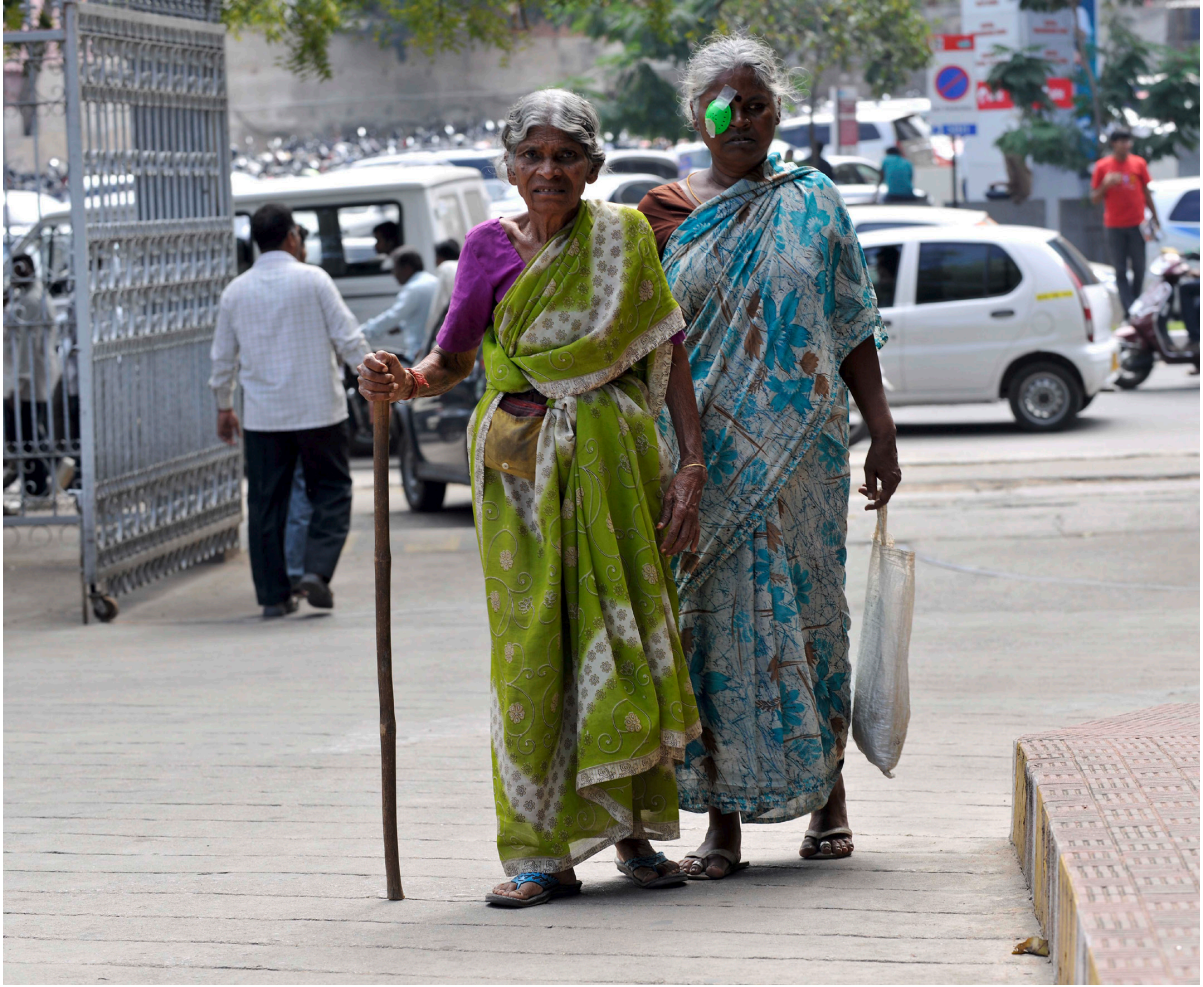


**Table – 2:** Technology & Quality

Sivaganga &Tirunelveli Surveys				
Vision Category	Presenting Visual Acuity		WHO Standard for Vision Outcomes	
	IOL (n=840)	Non-IOL (n=989)	Presenting	Best Corrected
**Normal (≥ 6/18)**	77.3%	49.3%	80%+	90%+
**Impaired (<6/18-≥6/60)**	18.0%	10.9%	15%+	5%+
**Blind (< 6/60)**	4.7%	39.8%	<5%	<5%

## Human Resource and Infrastructure:

The scarcity of trained ophthalmic manpower is aggravated by the fact that they tend to be based in large urban centres. This in turn dictates the location of eye hospitals and other eye care infrastructure as well. An earlier assessment of distribution in India showed that over 57% of the ophthalmologists were based in 56 cities which accounted for only 11% of the population.^4^ Conscious of this urban concentration of eye care services, programmes emerged to reach out to the rural areas essentially through eye camps. However, the reach and impact of this approach has been limited.^5^

## Technology and Quality:

Most technologies tend to be developed in the West and are priced to be relevant to those markets. Some technologies, like an intra-ocular lens offer a dramatically better outcome and quality of vision. The studies done in the 1990's showed that presenting visual outcome in the aphakic eyes (non IOL) was categorised as blind (vision less than 6/60) in 40% of the eyes, while in the same survey it showed that amongst the pseudophakic eyes (with an IOL implant) the blindness rate was as low as 4.7%.^6^,^7^,^8^

Such vast variations in the quality of outcome affect demand and fuels the dynamics of inequity. In this instance the inequity of who got a better outcome was brought about by the high price of the imported lenses. In the case of IOL, this was addressed in India and Nepal, which set up several IOL manufacturing factories and priced the IOLs to suit the economies of South Asian countries. Bringing about such equities has been possible only in a few instances like IOLs, sutures and some pharmaceuticals. In many other areas, inequities in quality driven by technology and their price continue to exist.

## Research and Evidence:

Though indirectly, research also seems to have played an unintended role in fuelling inequity. When one looks at the data around research and publication, it shows that over 92% of peer reviewed publications emanate from developed countries, which account for 10% of global blindness. The developing countries which account for over 90% of blindness contributed to less than 8% of the publications.^9^,^10^ The local knowledge and evidence that emerge from research are fundamental for effective design of interventions and services. Conversely the lack of such evidence based design leads to sub-optimal delivery of care and unintentionally results in inequities.

When one looks at the macro design of eye care in developing countries, one sees that the overarching and in some instances, exclusive attention is given to hospital infrastructure. This has been largely at secondary level, essentially to offer treatment to those who present themselves. This is a model that is designed to be reactive to demand. This is quite appropriate to the western world, where most people in need of eye care have the wherewithal and would seek it. However, in developing countries the design has to be more proactive to stimulate demand. Significant emphasis has to be on provision of appropriate eye care service at primary level recognizing the realities of the rural-urban divide, scarcity of skilled human resource and access challenges.

Paying capacity is another significant factor in developing countries where most of the care is financed through out of pocket payments; unlike in the West where the State or near universal insurance mechanism eliminates the affordability barrier. In hindsight, eye care systems in developing countries should have been built on a robust foundation of primary eye care. The evidence for this is just emerging and so is the establishment of primary eye care.

## Conclusion & Suggestions:

Inequity should not continue to be a by-product of population studies ora mere means of explaining the growing backlog. It has to influence the design of eye care services by making “inequity” the central concern. Such re-design should happen at both operational care level and at the broader eco-system level. National policies should encourage local evidence and research. Necessary capabilities will need to be developed and funding provided. Likewise regulations and policies should allow for easy access to cost-effective technologies as well as encourage local development. Focus should be to draw strategies and interventions to reach the unreached population and thereby eliminate inequality in eye care.
